# Enhancing LoRaWAN Security through a Lightweight and Authenticated Key Management Approach

**DOI:** 10.3390/s18061833

**Published:** 2018-06-05

**Authors:** Ramon Sanchez-Iborra, Jesús Sánchez-Gómez, Salvador Pérez, Pedro J. Fernández, José Santa, José L. Hernández-Ramos, Antonio F. Skarmeta

**Affiliations:** 1 Department of Information and Communications Engineering, University of Murcia, 30100 Murcia, Spain; jesus.sanchez4@um.es (J.S.-G.); salvador.p.f@um.es (S.P.); pedroj@um.es (P.J.F.); josesanta@um.es (J.S.); jose-luis.hernandez-ramos@ec.europa.eu (J.L.H.-R.); skarmeta@um.es (A.F.S.); 2 European Commission, Joint Research Centre, 21027 Ispra, Italy

**Keywords:** LoRaWAN, Internet of Things, security, EDHOC, key management

## Abstract

Luckily, new communication technologies and protocols are nowadays designed considering security issues. A clear example of this can be found in the Internet of Things (IoT) field, a quite recent area where communication technologies such as ZigBee or IPv6 over Low power Wireless Personal Area Networks (6LoWPAN) already include security features to guarantee authentication, confidentiality and integrity. More recent technologies are Low-Power Wide-Area Networks (LP-WAN), which also consider security, but present initial approaches that can be further improved. An example of this can be found in Long Range (LoRa) and its layer-two supporter LoRa Wide Area Network (LoRaWAN), which include a security scheme based on pre-shared cryptographic material lacking flexibility when a key update is necessary. Because of this, in this work, we evaluate the security vulnerabilities of LoRaWAN in the area of key management and propose different alternative schemes. Concretely, the application of an approach based on the recently specified Ephemeral Diffie–Hellman Over COSE (EDHOC) is found as a convenient solution, given its flexibility in the update of session keys, its low computational cost and the limited message exchanges needed. A comparative conceptual analysis considering the overhead of different security schemes for LoRaWAN is carried out in order to evaluate their benefits in the challenging area of LP-WAN.

## 1. Introduction

Privacy and security have become two prominent fields of study within the Internet of Things ecosystem. With the advent of this novel paradigm, it is envisioned that a great amount of elements and objects participating in our daily life will be connected to the Internet in order to transmit actual measurements in real time. Although this fact will enable the development of a plethora of services with the aim of improving our quality of life, it is clear that several concerns related to the privacy and security of these data arise. Big efforts have been devoted to securing the cloud platforms that process and store the data retrieved by end-nodes. However, the aerial part of data transmissions are the weakest part of this chain, especially when they make use of highly constrained radio access technologies, in terms of bandwidth, data rate and packet size. This is motivated because these kinds of technologies tend to heavily reduce the overhead in the network, so the employed security schemes should be notably simplified. This is the case of Low-Power Wide-Area technologies (LP-WAN), which have greatly attracted the attention of both academia and industry recently. The interest in LP-WAN technologies has been motivated by their outstanding features that will push them toward being adopted as an extensively-used radio access technology in the near future. These characteristics are related to the long range of more than 10 km attained in rural areas and the very low power consumption that allows end-device battery lifetimes of more than five years [1].

In particular, in this work, we focus on one of the most relevant LP-WAN solutions, called Long Range Wide Area Network (LoRaWAN). This radio technology provides high grades of adaptability to user needs by tuning its Physical (PHY) layer parameters. By this characterization, LoRaWAN can be employed in many scenarios and under several propagation conditions [2]. In addition, LoRaWAN makes use of license-free frequency bands, so its adoption might be widely generalized soon. This will become a real issue related to the spectrum saturation if hundreds or thousands of nodes need to share wireless media at the same time. This situation is aggravated considering the low data-rate of the transmissions (from 250 bps–50 Kbps in LoRaWAN), which lead to a very high Time-on-Air (ToA) of the transmitted packets. Thus, the longer the packet length, the greater the transmission duration, so the probability of collisions or interference-related issues clearly grows. As the use of robust security mechanisms usually implies the addition of extra headers and long payloads, LP-WAN platforms tend to reduce the overhead introduced when securing the communication. Besides, end-nodes are usually highly constrained in terms of computation capabilities and power consumption, so they are not able to perform complex operations that are usually required by strong security schemes.

In the case of LoRaWAN, each end-device must be personalized and activated to communicate through the network. In particular, the LoRaWAN specification [3] defines two activation modes: Over-The-Air Activation (OTAA) and Activation By Personalization (ABP). The former assumes the existence of a pre-shared key (Application Key (AppKey)), which is used to derive two session keys (Application Session Key (AppSKey) and Network Session Key (NwkSKey)) through a join procedure. However, LoRaWAN does not define any key management or update mechanism for these session keys. Indeed, an end-device is forced to launch the join procedure every time session keys need to be updated. In ABP, this issue is exacerbated since both session keys must be manually configured on the end-device. This approach is highly inflexible and introduces cumbersome administrative tasks during the initial network deployment, representing an unfeasible solution for large-scale scenarios.

In order to address this problem, this work proposes the application of a lightweight mechanism for key management, so that end-devices are enabled to update session keys in an efficient and authenticated way. Our approach follows recent Internet Engineering Task Force (IETF) standardization proposals; in particular, it is based on the use of Ephemeral Diffie–Hellman Over COSE (EDHOC) [4]. Compared with widely-used protocols for the establishment of security associations, such as the Internet Key Exchange (IKE) [5] or Transport Layer Security (TLS) approaches [6,7], EDHOC combines the flexibility and strength of the use of public key cryptography, with the efficiency and lightness provided by Concise Binary Object Representation (CBOR) Object Signing and Encryption (COSE) [8]. In this way, LoRaWAN session keys can be derived from ephemeral cryptographic material by using public key cryptography, in order to provide Perfect Forward Secrecy (PFS) [9], while the network overhead is reduced. To the authors’ knowledge, this is one of the first real implementations and evaluations of EDHOC in such a constrained scenario. Thereby, the main contributions of this work are the following:

1. An exploration of the security vulnerabilities of LoRaWAN in the area of key management.
2. An analysis of alternative schemes to the ones proposed by LoRaWAN.
3. The proposal of a lightweight solution based on the EDHOC protocol and integrated in an IPv6-capable LoRaWAN architecture.
4. A comparison of the overhead introduced by the proposal as compared with the main competing approaches.

The rest of the paper is organized as follows. Section 2 explores the related literature addressing security issues in IoT constrained systems, with a strong emphasis on key management issues. Section 3 presents LoRaWAN and its activation mechanisms in which security aspects are considered. Section 4 focuses on the security issues of LoRaWAN and proposes alternative security protocols to cover the identified flaws. On the basis of this review, Section 5 proposes a reference LP-WAN architecture with a lightweight and authenticated key management approach based on EDHOC. This solution is evaluated in Section 6, comparing the different considered proposals in terms of protocol overhead. Finally, Section 7 concludes the work, presenting the most significant findings.

## 2. Related Work

The application of security and privacy mechanisms in IoT scenarios still remains as one of the main barriers to be overcome in the coming years for the realization of new services and applications [10]. Under the vision of an IP-enabled IoT, the interconnection of constrained physical devices through the use of Low power and Lossy Networks (LLNs) requires the adaptation of current mechanisms and protocols that are commonly used nowadays. Beyond the significant interest from academia and industry, from the perspective of standardization, the IETF has defined specific working groups in order to address such aspects. On the one hand, the Constrained RESTful Environments (CoRE) WG (https://datatracker.ietf.org/wg/core/about/) encompasses different initiatives, in order to fit communication protocols and data formats in IoT environments with tight resource constraints. Indeed, the Constrained Application Protocol (CoAP) [11], which is widely used nowadays, was defined within the scope of CoRE. On the other hand, the Authentication and Authorization for Constrained Environments (ACE) WG (https://datatracker.ietf.org/wg/ace/about/) is focused on the development of lightweight authentication and authorization mechanisms, through the adaptation of well-known approaches to constrained devices and networks. Additionally, the CBOR Object Signing and Encryption (COSE) [8] can be considered as the evolution of JavaScript Object Notation (JSON) Object Signing and Encryption (JOSE) [12] with a more lightweight and efficient representation format based on CBOR. COSE represents the main building block of EDHOC, which is an ongoing effort within ACE.

Given the recent appearance of LP-WAN technologies, the literature especially focused on security issues in this area is still limited [13]. In the case of LoRaWAN, some works have recently explored the potential vulnerabilities of the security mechanisms adopted by this technology [14]. In particular, the work in [15] focused on evaluating the security of the LoRaWAN joining process with special emphasis on the nonce generation during this phase. The authors found this scheme to be prone to Denial of Service (DoS) attacks by the regeneration of an already used nonce by an end-device. Jamming attacks were also considered, and the system robustness was evaluated, showing its vulnerability to this kind of malicious event. In turn, the work in [16] presented a more descriptive analysis of the security risks suffered by LoRaWAN networks. The authors found that the long LoRaWAN packet’s ToA is highly attractive to potential attackers because it facilitates jamming, replay or wormhole attacks. A physical attack was also performed on an end-node, extracting the pre-shared key from it, hence leaving the device open to hijacking. However, these works did not propose any concrete security scheme in order to face the cited weaknesses. In addition, the work in [17] carried out an analysis of the main LoRaWAN security aspects and proposed an extended architecture by adding a typical certificate-based approach and transport layer security. While this solution could help as a complementary approach for the development of high-level services, key management aspects are not considered at the LoRaWAN level.

From the set of the main LoRaWAN security issues, key management represents a major obstacle that deserves further attention. On the one hand, LoRaWAN does not provide any update mechanism for session keys. This way, an end-device could employ the same cryptographic material throughout its lifetime. Therefore, if these keys are leaked, an attacker could access all the information previously transmitted by that end-device. Furthermore, according to the LoRaWAN specification, the network server is responsible for generating session keys, so it becomes a single point of failure for security aspects. These issues have motivated the development of different works in order to enhance key management in LoRaWAN. In this direction. Kim et al. [18] proposed a device-to-device link establishment scheme for LoRaWAN. In this approach, the network server is responsible for managing and distributing the same cryptographic material to two devices, by defining new messages to be exchanged between the server and the end-devices. From this material, both devices generate the same key that is used for a secure communication. The proposed approach is based on the extension of the work presented in [19] for device-to-device communications in LoRaWAN. However, while these works provide valuable insights for possible LoRaWAN extensions, they do not address the aspects related to key management between end-devices and the server side.

The work in [20] proposed to add proxy nodes to the LoRaWAN architecture to alleviate the burden of key management on end-devices. Their approach is also based on the use of a reputation system to select the most trustworthy proxy nodes. Nevertheless, they do not provide evaluation results to demonstrate its feasibility, and consequently, the application of the proposed approach in LoRaWAN scenarios remains unclear. In addition, the work in [21] proposed an improved activation scheme for LoRaWAN to enhance key management aspects of the current specification. It separates the key management between the network server and the application server, and the end-device maintains two pre-shared keys; an AppKey with the application server and a Network Key (NwkKey) with the network server. Unlike the LoRaWAN specification, they propose each session key to be derived from the keys used in previous sessions (i.e., instead of the static AppKey and NwkKey keys). However, through such a key update approach, perfect forward secrecy cannot be provided, since session keys are obtained from keys previously used. It should be noted that while the recent LoRaWAN 1.1 specification [22] already considers similar enhancements, session keys are always derived from the static NwkKey and AppKey.

In this work, alternative security protocols, such as Datagram Transport Layer Security (DTLS), IKE and EDHOC, are evaluated as potential approaches for key management in LoRaWAN networks. Based on our analysis, we propose a security extension based on the novel IETF’s draft EDHOC, the messages of which are carried through the CoAP protocol. The resulting approach is considered as an appropriate mechanism, due to the provided trade-off between lightness and strong security properties to be applied to IoT constrained scenarios. This way, unlike recent approaches to key management in LoRaWAN, our proposal uses the cryptographic material derived at the application layer to update LoRaWAN session keys, to come up with a lightweight and flexible approach for key management in these networks.

## 3. LoRaWAN

This section presents the base LoRaWAN features and describes the currently supported security mechanisms, which foster the discussion carried out in the next sections.

### 3.1. Overview

LoRaWAN is a long-range low-power transmission technology that has had great attention devoted to it in recent times. It is supported by the LoRaWAN Alliance, which is formed by important companies such as Cisco and Semtech, among many others [23]. It is well defined as a two-layered architecture that determines the transmission (PHY) level with LoRa and the Medium Access (MAC) layer with LoRaWAN. The former, LoRa, is a proprietary modulation scheme defined by Semtech that employs a Chirp Spread Spectrum (CSS) modulation with the aim of strengthening the communication reliability. However, this modulation provides relatively low transmission rates of about a few kilobits per second. This limitation, together with the fact that LoRaWAN makes use of unlicensed bands, leads to a stringent restriction in terms of the transmitted packet size in order to reduce the ToA of each packet. Aiming at providing the communication system with some adaptation capabilities, LoRa modulation presents three configurable parameters that notably determine the characteristics of the transmissions in terms of maximum link distance, communication robustness, etc. These parameters are: Bandwidth (BW), Coding Rate (CR) and Spreading Factor (SF). While the first one is usually fixed to 125 kHz, the others are often tuned to adapt the transmission characteristics to the propagation and environmental conditions. The CR determines the amount of redundant information included in each packet for error recovery purposes, and the SF permits increasing the communication range and its robustness at the expense of reducing the data rate. On the other hand, as previously mentioned, LoRaWAN defines the MAC layer of the stack, enabling highly valued characteristics for IoT applications, such as synchronization schemes, acknowledgment mechanisms or a security suite, among others. Additional details about LoRaWAN can be found in [2].

### 3.2. Security Features

From a security perspective, LoRaWAN implements a cryptographic mechanism based on AES-128 working in counter (CTR) mode. It is based on a pre-shared key from which two additional keys are derived for securing the session. Before performing this derivation process, end-devices must be activated in order to gain connectivity through a specific LoRaWAN network. Otherwise, the transmitted frames are silently discarded by the network server. In order to consider an end-device as activated, it must own a valid copy of the following data, an end-Device Address (DevAddr), a Network Session key (NwkSKey) and an Application Session Key (AppSKey). Depending on the adopted joining scheme, these data may be manually stored in the device or they might be obtained through the radio link. Besides, no update period is defined for these keys, which might be identified as a security vulnerability, as is examined in the next sections. In this paper, this particular fact and how the security solution adopted in LoRaWAN consequently lacks flexibility in order to quickly react to security attacks are discussed. Besides, the management efforts related to the security-scheme setup are considerably high as a manual intervention is needed for its configuration.

#### 3.2.1. Activation by Personalization

In this case, the authentication data are hard-coded into the device before it begins the communication with the network; hence, no join procedure is required. These data tie the end-device to a particular LoRaWAN network, as they contain the LoRaWAN Network Identifier (NwkId), the Network Address (NwkAddr) of the device and the cryptographic session keys. Thus, the end-device is not allowed to communicate with other LoRaWAN networks while these values remain the same.

#### 3.2.2. Over-The-Air Activation

**Figure 1:**
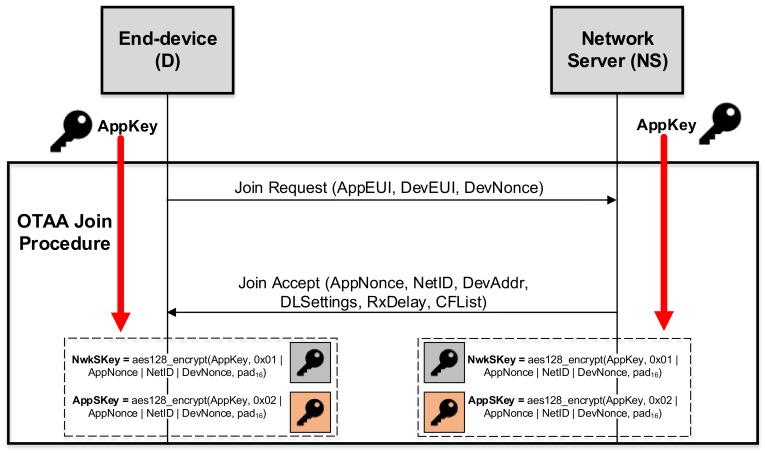
Overview of the OTAA join procedure.

By considering this strategy, a well-defined join procedure is performed by each end-device with the aim of obtaining the needed cryptographic material to enable the secure connection to a specific network. Figure 1 shows an overview of this activation mode. In order to start the joining process, the end-device must own the following information: an End-Device Identifier (DevEUI), an Application Identifier (AppEUI) and an Application Key (AppKey). The DevEUI uniquely identifies the end-device, while the AppEUI identifies an entity that is able to process the end-device’s join-request. In addition, the AppKey is an AES-128 key employed in the subsequent session key derivation process. Thereby, as an initial step, the end-device sends a join-request message that contains the AppEUI, DevEUI and DevNonce. Note that the join-request message is not encrypted; therefore, a DevNonce value is used in order to avoid replay attacks. The data contained in this first message enable the derivation of the two session keys, namely, NwkSKey and AppSKey, which is performed as follows:

$$NwkSKey=aes128\_encrypt(AppKey,0x01|AppNonce|NetID|DevNonce|pad_{16})\;AppSKey=aes128\_encrypt(AppKey,0x02|AppNonce|NetID|DevNonce|pad_{16})$$

Finally, if the process is correct, the network server responds with a join-accept message in order to close this transaction and confirm the end-device authentication; this message is already encrypted using the AppKey. As already mentioned, LoRaWAN does not provide any mechanism to update these session keys; consequently, an end-device is required to carry out a new activation process in order to refresh such material. Even in this case, the new session keys are always derived from the static and pre-shared AppKey key. In contrast, our approach is based on the use of ephemeral cryptographic keys that are employed to generate new NwkSKey and AppSKey keys each time it is required.

## 4. Enhancing LoRaWAN Key Management

This section presents some of the main vulnerabilities found in the LoRaWAN security scheme. Thereafter, specific well-known security schemes are studied as alternatives and their pros and cons related to their integration in the LoRaWAN stack are identified.

### 4.1. Identified Deficiencies

As stated above, the security implemented in off-the-shelf LoRaWAN systems is based on a pre-shared key, the so-called Application Key (AppKey), from which two additional keys are derived: the Network Session Key (NwkSKey) and the Application Session Key (AppSKey). The latter is employed to protect the packet payload, so it is shared just between both extremes of the communication to ensure the confidentiality of these data. In turn, the NwkSKey is shared among the end-device and the networking elements in order to permit routing tasks and sanity checks. The establishment of such keys depends on the selected joining scheme, which permits end-devices to be authenticated by the network.

Two different security procedures have been previously detailed, namely Over-The-Air Activation (OTAA) and Activation By Personalization (ABP). During the OTAA, which is the usually recommended scheme, a message exchange including cryptographic material is performed between the end-device and the network server. By using this material and the AppKey, both the NwkSKey and the AppSKey are derived. After this step, communications are secured by using the derived keys. This process is carried out when the device connects for the first time to the network or in case of connectivity loss. However, no updating period of these keys is defined.

Even more static, the ABP scheme proposes to hard-code the two session keys, so their updating process needs manual intervention from the network manager. Therefore, end-devices do not perform any periodic update of such keys (static session keys), which means that the probability of the NwkSKey and the AppSKey being compromised is greater since they are constantly used to protect the communications. Thus, if an attacker succeeds in obtaining these session keys, all the encrypted information exchanged between the end-device and the network server will be accessible during a long period of time until the attack is detected.

It is interesting to mention that the newest release of LoRaWAN, i.e., LoRaWAN 1.1, although increasing the complexity of its security mechanisms, e.g., a wider set of session keys is employed, the deficiencies described above remain the same as the session keys are still derived from the non-removable NwkKey and AppKey.

### 4.2. Potential Alternatives

Cybersecurity issues caused by the use of static NwkSKey and AppSKey motivate the need to incorporate a session key update mechanism into LoRaWAN to ensure an adequate protection of the information shared between the end-device and the network server. In addition, such a key update mechanism should be periodically executed according to different practical aspects of the network, e.g., the degree of sensitivity of the exchanged information. In this sense, there are different protocols that may be considered as potential alternatives to design a suitable key management approach. In particular, we assess the use of IKEv2 [5], DTLS [7] and EDHOC [4].

#### 4.2.1. DTLS

Nowadays, CoAP represents the main protocol for the application layer to be considered in IoT constrained scenarios. From the security point of view, it specifies a binding to DTLS for securing communications involving resource-constrained devices [11]. Therefore, this protocol emerges as a candidate to provide security in IoT environments, enabling different mechanisms of authentication, key exchange and application data protection. DTLS is based on TLS [6], thus being a layered protocol. In particular, the Record protocol is used to encapsulate the messages of the Handshake, Alert, Change Cipher Spec and Application Data protocols. From these, the Handshake protocol allows one to establish certain security parameters between two devices (e.g., cryptographic algorithms or authentication schemes), so that they are able to calculate symmetric keys, in order to protect communications between them. Furthermore, the Alert protocol allows one to transmit certain anomalous situations, while the Change Cipher Spec protocol allows one to notify that the next exchanged messages will be protected by the just-negotiated algorithms and symmetric keys. Finally, the Application Data protocol enables the exchange of encrypted data between two devices once the handshake process has been successfully completed.

Accordingly, an approach based on the use of the DTLS Handshake protocol could be considered as an alternative to derive and update cryptographic material. It should be noted that the use of DTLS is intended to protect data from the application layer. However, instead of protecting the upper layer, in this case, the cryptographic material derived from the handshake would be used by LoRaWAN. In particular, the keys derived from the handshake protocol would be used to update the session keys (NwkSKey and AppSKey) in LoRaWAN. For this purpose, the end-device and the network server should select a cipher-suite that fits into the security mechanisms already established in this network. For example, in order to maintain the use of the AppKey to be compliant with LoRaWAN specification, the cipher-suite TLS_PSK_WITH_AES_128_CCM_8 [24] could be considered. This way, the AppKey can be used as the Pre-Shared Key (PSK) for authentication purposes. However, this cipher-suite does not allow one to achieve Perfect Forward Secrecy (PFS) [9] because it does not use the Diffie–Hellman algorithm with Ephemeral keys (DHE) to compute session keys. Therefore, if one of these session keys is compromised, encrypted information with previous keys could be accessible in an unauthorized way. In addition, it should be pointed out that the choice of the DTLS handshake as the mechanism for updating the NwkSKey and AppSKey would require a heavy message exchange between the end-device and the network server, according to the DTLS specification. Therefore, if the LoRaWAN network is made up by a large number of end-devices, the use of this handshake protocol can lead to a high network overload.

#### 4.2.2. IKEv2

**Figure 2:**
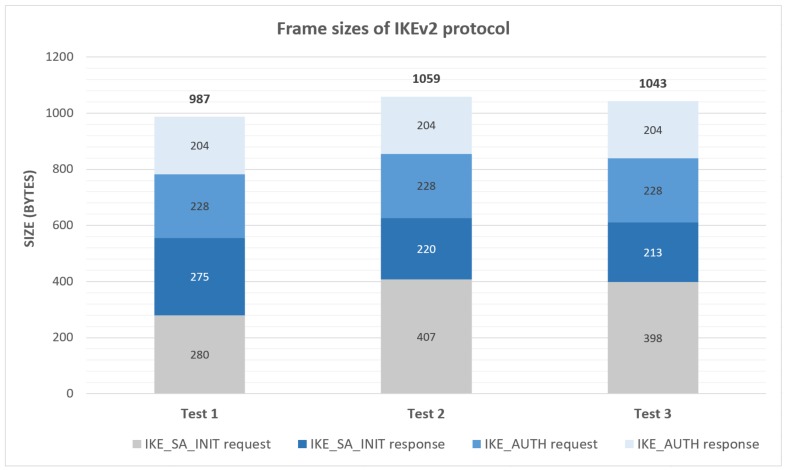
Frame sizes of IKEv2 protocol only taking into account IKEv2 header and payload.

IKEv2 can be used as a generic key-management service in order to generate and frequently refresh keying material between client and server. In this case, the authentication can be performed by using a pre-shared key, as well as certificates. IKEv2 mechanisms make use of the Diffie–Hellman algorithm, so they achieve Perfect Forward Secrecy (PFS) protection. The Diffie–Hellman algorithm performance cost depends on the chosen cipher-suite. For this type of strongly constrained scenario, an elliptic curve group [25] can be selected to reduce the cost and processing time. In addition, IKEv2 is based on message exchanges (request and response) and uses one exchange to establish an initial security association only to protect subsequent IKEv2 messages and another one to negotiate an initial security association that protects the transmitted data using Internet Protocol Security (IPsec). The process comprises four messages in total to perform an initial security association (IKE_SA_INIT and IKE_AUTH exchanges). However it only needs one extra exchange (CREATE_CHILD_SA exchange) for each keying update of an existing association or for each new IPsec association establishment. Hence, this protocol starts to become more convenient when then number of security associations to be established by the peer is high. Nevertheless, an inconvenience of the IKEv2 protocol is its message sizes, which exceed the supported limit for LoRaWAN messages at lower data-rates. As a proof of that, we have performed the initial exchanges three times to check the message sizes. In Figure 2, you can see the results. The sizes slightly oscillate due to the variable size of the IKE_SA_INIT nonces. In this case, we have used PSK-based authentication and elliptic curve (Group 19) in order to be closer to the typical cypher-suits used in constrained environments. These sizes exceed the maximum length of LoRa messages, requiring fragmentation, with the extra complexity associated. Concretely, the maximum LoRa payload lengths are the following, SF7–SF8: 255 B, SF9: 128 B, and SF10–SF12: 64 B.

Another inconvenience is that IKEv2 implementations are thought to be used in conjunction with the IPsec stack (network layer), and they are not prepared to provide the generated keying material to third party applications (application layer).

#### 4.2.3. EDHOC

As mentioned above, the CoAP specification proposes the use of DTLS to secure communications in networks made up of resource-constrained devices. However, there are situations in which transport layer security is not sufficient to achieve end-to-end security [26], or security aspects need to be provided independently of the technology or protocol being used. In this sense, as [4] specifies, EDHOC can be a security alternative to the DTLS handshake protocol to cope with this issue, also considering the limitations of IoT devices in terms of processing capacity, memory, storage and power consumption [27]. EDHOC is a lightweight key exchange protocol that allows the establishment of symmetric keys between two devices. Like IKEv2 and DTLS, it is based on a SIGMA (SIGn-and-MAc) protocol variant [28], specifically the SIGMA-I variant, implementing the Elliptic Curve Diffie–Hellman algorithm (ECDH) [29] with ephemeral keys to provide PFS. In addition, EDHOC establishes two authentication modes based on public key (i.e., raw public keys and certificates) and Pre-Shared Keys (PSK). This way, the authentication and the key generation processes remain independent of each other. Moreover, EDHOC defines a three-message exchange, which can be embedded in different protocols (e.g., CoAP). Furthermore, these EDHOC messages are encoded following the concise binary object representation [30] and protected by CBOR Object Signing and Encryption (COSE) [8], so that they can be processed and verified efficiently by IoT devices with constrained resources.

**Figure 3:**
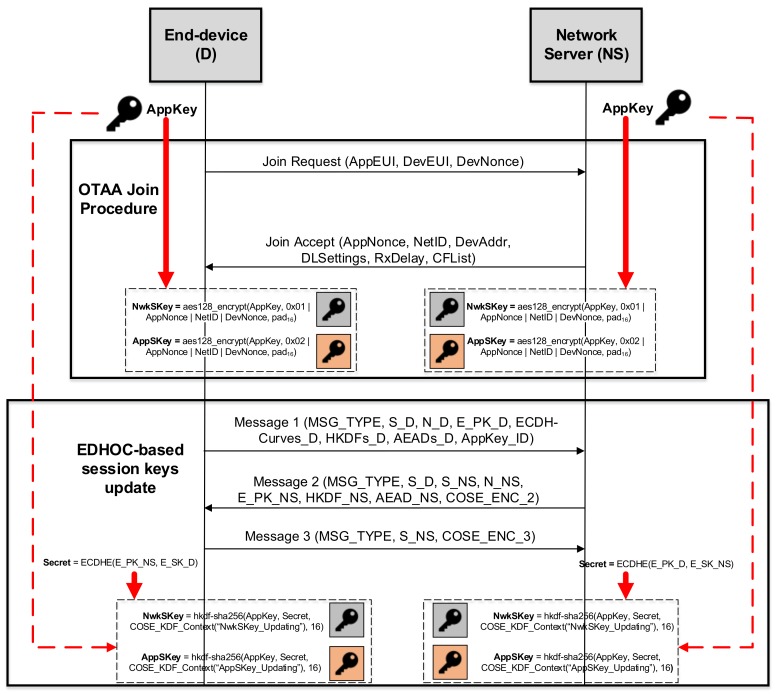
Overview of the EDHOC integration to enhance LoRaWAN key management.

In order to show the integration of EDHOC with LoRaWAN as the session key-updating mechanism, Figure 3 shows a high-level view of the required process. It should be noted that EDHOC is intended to be used once the end-device is activated, in order to update the session keys. Note that we select the authentication mode based on a pre-shared key so that the AppKey acts as the PSK. According to this scheme, EDHOC only requires the exchange of three messages between the end-device and the network server to update the NwkSKey and AppSKey. Therefore, it entails a lower network overload than the DTLS handshake protocol and IKEv2 when a single security association is needed. For the sake of clarity, we focus the description on the fields that are closely related to the key management approach. In particular, Message 1 is sent by the end-device, including (among other parameters) both its ephemeral public key (E_PK_D) and an identifier (AppKey_ID) associated with the AppKey used to carry out the authentication process. Message 2 includes the network server ephemeral public key (E_PK_NS), as well as a COSE object (COSE_ENC_2) that authenticates it and protects the integrity of Messages 1 and 2. Finally, Message 3 is used for end-device authentication and ensuring the integrity of all messages by another COSE object (COSE_ENC_3). It should be pointed out that, as EDHOC messages are encoded following the COSE and CBOR standards, the size of such messages is more reduced than with other representation formats, such as JSON, favoring their processing by constrained end-devices. When the EDHOC message exchange has successfully finished, the shared secret (Secret) is computed. For this purpose, the end-device runs the Diffie–Hellman algorithm with its ephemeral secret key (E_SK_D) and the E_PK_NS. Similarly, the network server runs such an algorithm with its ephemeral secret key (E_SK_NS) and E_PK_D. Once both devices obtain the Secret, they apply a key derivation function to get the corresponding session keys. In particular, the HMAC-based Extract-and-Expand Key Derivation Function (HKDF) [31] is proposed by the EDHOC to perform this derivation operation. Thus, the keys’ computation is performed as follows:

$$NwkSKey=hkdf-sha256(AppKey,Secret,COSE\_KDF\_Context{(}^{\prime \prime }NwkSKey\_Updating^{\prime \prime }),16)\;AppSKey=hkdf-sha256(AppKey,Secret,COSE\_KDF\_Context{(}^{\prime \prime }AppSKey\_Updating^{\prime \prime }),16)$$

where the *COSE_KDF_Context(AlgorithmID)* structure is defined by following the standards of [4,8]:

COSE_KDF_Context(AlgorithmID)=(AlgorithmID,(null,null,null),16,″″,sha256(sha256(Message1|Message2)|Message3))

As is shown, the HKDF makes use of SHA-256 as the hash function including the following parameters: the static and pre-shared AppKey, the previously computed Secret, a *COSE_KDF_Context(AlgorithmID)* structure and the length of the derivated key (a 16-byte length in this case according to the length of AppSKey and NwkSKey). Note that such a *COSE_KDF_Context(AlgorithmID)* structure is different for each session key. Specifically, the AlgorithmID parameter establishes “AppSKey_Updating” and “NwkSKey_Updating” for the AppSKey and NwkSKey, respectively. This way, the session key update mechanism allows one to obtain AppSKey and NwkSKey independently of each other.

## 5. Proposed Architecture

In light of the previous discussion, it can be seen that both DTLS handshake and IKEv2 present problems in carrying out the update of the NwkSKey and the AppSKey in LoRaWAN, as they were not designed to work in this highly constrained scenario. For that reason, EDHOC has been considered in a new proposal for enabling the exchange of cryptographic material in LP-WAN scenarios and, in particular, in LoRaWAN.

**Figure 4:**
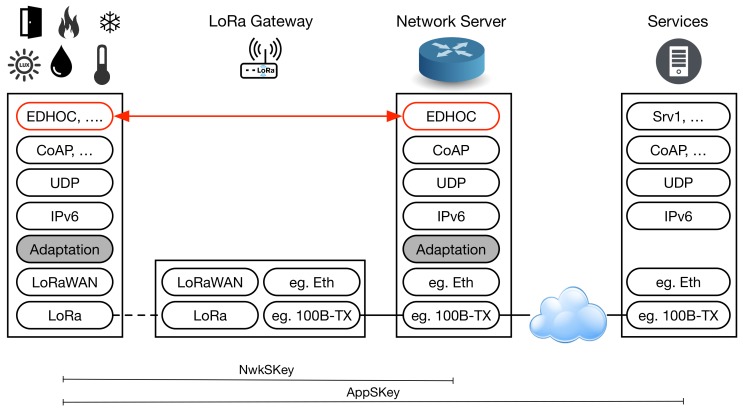
Architecture of the proposed security solution.

The general full-stack architecture proposed can be seen in Figure 4. EDHOC is used to derive the session keys, namely NwkSKey and AppSKey, that were previously obtained with LoRaWAN OTAA, but now improving flexibility in the key management as indicated above.

The communication stack presented in Figure 4 is based on the work initially presented in [32], which considered the transmission of Internet Protocol Version 6 (IPv6) datagrams over LoRa. As can be seen, application-level packets are encapsulated into User Datagram Protocol (UDP) datagrams. This transport protocol is assumed in order to diminish the network overload over the constrained LP-WAN link and because connection-oriented services are not common in these networks. Then, regular IPv6 datagrams are adapted to be sent over the given LP-WAN technology. For this, the Static Context Header Compression (SCHC) algorithm [33] is used. Finally, the compressed IPv6 packet is sent as a packet payload, in a physical frame through the LP-WAN (LoRa) link. Thereafter, the packet traverses the LoRa gateway to reach the network server, where the previous IPv6 adaptation is reverted. The original IPv6 packet is then forwarded to another network, probably traversing the Internet, until reaching the final IPv6 destination node, which implements the application level of the concrete service. Of course, this process is bi-directional, and the end-nodes are able to receive and de-compress IPv6 compressed packets, as well. In the architecture, the EDHOC peers are included in the end-device and the network server. Prior to any communication with any application server, an EDHOC negotiation is needed to derive the session keys. Then, the common LoRaWAN security procedure is followed, using the NwkSKey for integrity protection and encryption of the LoRaWAN MAC commands and the AppSKey for encrypting application-level messages.

**Figure 5:**
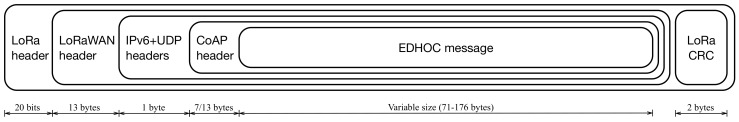
LoRa frame finally created to transport EDHOC messages.

In this proposal, similar SCHC rules to the examples shown in [33] are employed. Hence, only the rule ID and application payload are sent over the constrained link. As a result, the IPv6 and UDP headers are compressed jointly in a one-byte field. The whole LoRa frame used to transport EDHOC messages is shown in Figure 5. The LoRa frame requires a header of 20 bits, which should be also preceded by a preamble that depends on the modulation chosen and it is not included in Figure 5. The LoRaWAN header including the required options to transport our EDHOC messages is 13 bytes, while the CoAP header is 13 bytes for requests and seven bytes for responses. The finally encapsulated EDHOC message has a variable size, depending on the type of message and the optional compression, as described in the next section.

We are in the development of a prototype of the stack described in Figure 4. In particular, our implementation of EDHOC uses the elliptic curve P-256 during the Diffie–Hellman algorithm, since this curve already provides the minimum security level recommended by the National Institute of Standards and Technology (NIST), that is 128-bit [34]. For the same reason, we selected AES with 128-bit symmetric keys as an Authenticated Encryption with Authenticated Data algorithm (AEAD) [35]. This algorithm, in addition to providing confidentiality to the information exchanged between the end-device and the network server, allows verifying its integrity and authenticity. Furthermore, EDHOC establishes the use of a Key Derivation Function (KDF) to compute the symmetric key shared between two IoT devices from the secret obtained by the Diffie–Hellman algorithm. The main goal of this function is to destroy existing algebraic relationships between different public keys that can lead to obtaining the same secret. This way, new computed symmetric keys are cryptographically strong [36]. In our case, we choose the HKDF function since it is recommended by NIST as a mechanism for key derivation through extraction-then-expansion [37], and additionally, the EDHOC specification forces its implementation. Finally, regarding the COSE and CBOR libraries used in our development, we use the GitHub project pointed out in the COSE specification (https://github.com/cose-wg/COSE-C).

## 6. Analysis of Solutions

The NwkSKey and AppSKey management in LoRaWAN is presented as a challenge to be addressed in order to ensure that information exchanged between the end-device and the network server is properly protected. In this sense, our proposal focuses on the incorporation of an EDHOC-based lightweight and authenticated mechanism to carry out the updating of such keys, considering the limitations of this type of network. In addition, unlike other previous works, such as the one already mentioned in Section 2 [21], our solution makes use of ephemeral cryptographic material with the purpose of providing PFS. This is a key aspect to improve the security of LoRaWAN networks since, if a previous key is compromised, only information protected under this key can be accessed, while the rest of the communications still remain secure. It should be pointed out that the solution proposed implies messages that can be still properly transmitted over LoRa. Accordingly, this section shows the overhead introduced by our solution in order to ensure its usage and validate its suitability to LoRaWAN networks.

With the purpose of demonstrating the advantages offered by the previous network architecture based on EDHOC, we present here an evaluation of the proposal and comparison with a DTLS-based approach. To the authors’ knowledge, this is the first work examining a real implementation of EDHOC in order to explore its introduced overhead. Given the strident computational requirements of IoT deployments, a detailed analysis of the message exchanges and sizes is addressed, in order to justify the selection of the most convenient security technology. In particular, we compare the DTLS handshake using the cipher-suite TLS_PSK_WITH_AES_128_CCM_8 with the EDHOC to show the overhead introduced by both protocols. Note that, in our approach, EDHOC messages are exchanged as payloads of the CoAP protocol [11], following the EDHOC specification [4]. Therefore, we refer to this protocol as CoAP/EDHOC. Additionally, it should be pointed out that IKE is not considered in this analysis, since it was not conceived of to operate in resource-constrained scenarios. Specifically, current specifications [5] do not define compact representation formats for the cryptographic material to be exchanged. Furthermore, recent proposals in the scope of the IETF ACE WG [38] already consider the use of EDHOC as an alternative to IKE for the establishment of IPsec Security Associations (SA) in constrained environments.

To carry out the tests, we have deployed the end-device on a PIC32MX795F512L with 80 MHz, 512 KB ROM and 128 KB RAM. Please observe that although this device may present higher computational and memory capacities than typical IoT end-nodes, in this work, we focus on the restrictions posed by the transmission technology rather than by the processing hardware. Similarly, the network server has been deployed on an Intel Core i5 with 2.7 GHz and 8 GB RAM. In addition, we have made use of software libraries to carry out these tests. Concretely, in the case of EDHOC, we have developed our own library. In the case of CoAP, we have made use of the erbium library (http://people.inf.ethz.ch/mkovatsc/erbium.php), and for DTLS, we have selected the tinyDTLS library (https://projects.eclipse.org/projects/iot.tinydtls).

**Figure 6:**
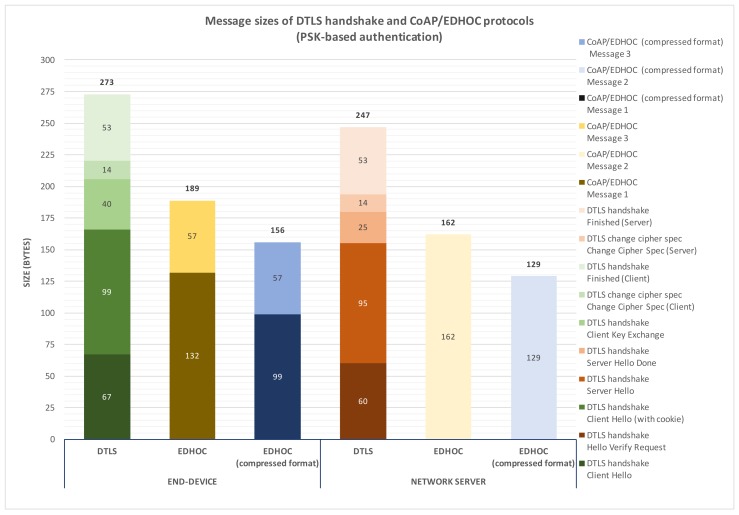
Message sizes of DTLS Handshake and CoAP/EDHOC protocols from the end-device and network server sides with PSK-based authentication.

As previously mentioned, the authentication mechanism used both by DTLS Handshake and CoAP/EDHOC in order to update the NwkSKey and the AppSKey is based on a key shared between the end-device and the network server to carry out the authentication process (PSK mode). Accordingly, Figure 6 shows the sizes of each message both of DTLS Handshake and CoAP/EDHOC to compute a shared secret from which the keys may be updated. According to the results, the end-device needs to send five messages when the DTLS Handshake protocol is employed, while in the case of CoAP/EDHOC, it only sends two messages. In addition, the overhead introduced by using CoAP/EDHOC from the end-device side is about 31% lower than by using DTLS Handshake. Additionally, the network server sends five messages with the DTLS Handshake protocol and one message when using CoAP/EDHOC. Attending to the message sizes, this means an overhead reduction of 35% as compared to DTLS. In general, as anticipated, this analysis demonstrates that EDHOC implies a lower number of messages and a reduced overhead as compared to DTLS Handshake as the mechanism of updating NwkSKey and AppSKey. Furthermore, and as shown in this figure, it should be noted that we have also tested the EDHOC protocol considering the compressed representation format for ephemeral public keys defined in [39]. By using such a compressed representation format, EDHOC message sizes are further reduced, which entails less overhead with respect to the DTLS Handshake protocol. Specifically, an overhead reduction of 43% from the end-device side and 48% from the network server side is achieved. Nevertheless, this representation format also leads to more processing time both by the network server and, especially, by the end-device due to it requiring additional operations to expand and retrieve ephemeral public keys. Therefore, its usage (or not) depends on different practical aspects related to the particular scenario.

**Figure 7:**
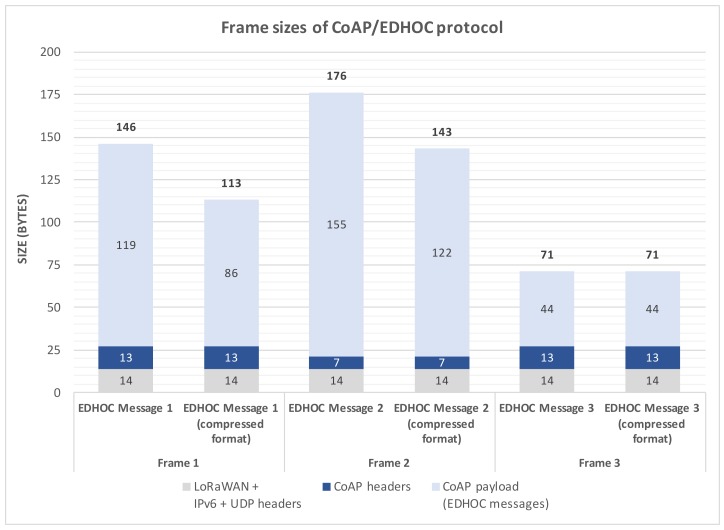
Sizes of MAC frames (LoRaWAN) including CoAP/EDHOC messages.

Attending to the final frames transmitted to the LoRa medium, Figure 7 shows the sizes of MAC frames that include the different CoAP/EDHOC messages when pre-shared keys used as the authentication mechanism. As previously mentioned, these messages must be exchanged between the end-device and the network server to compute the shared secret.

It should be pointed out that we compress the IPv6 and UDP headers to one byte by using the procedure summarized in Section 5, while the LoRaWAN header is held in 13 bytes. Furthermore, the total size of CoAP headers is variable, being 13 bytes for CoAP requests (Messages 1 and 3) and seven bytes for CoAP responses (Message 2). In addition, note that the EDHOC Messages 1 and 2 include the ephemeral public keys corresponding to the end-device and the network server. In this sense, in order to represent these public keys, we make use of both non-compressed and compressed representation formats, as previously mentioned. With the non-compressed representation format, ephemeral public keys are represented by *x* and *y* coordinates of 32 bytes in length each. Accordingly, it can be seen that Messages 1 and 2 require LoRa payloads of 146 and 176 bytes. On the other hand, when the compressed representation format is employed, ephemeral public keys are encoded by using only the *x* coordinate of 32 bytes in length, while the *y* coordinate must be recomputed. Hence, Messages 1 and 2 entail LoRa payloads of 113 and 143 bytes, respectively. Finally, Message 3 requires a payload of 71 bytes in both cases since this message does not include ephemeral public keys. These values should be conscientiously considered in LoRaWAN systems due to their communication restrictions.

As stated before, an important limitation of LoRa technology is its reduced transmission data-rate. This fact leads to long transmission times (ToA) that severely limit the maximum packet length that is allowed to be transmitted over the LoRa link. As previously mentioned, LoRa’s SF parameter might be tuned, which notably determines the wireless link robustness (please, see Section 7 of [3]). Therefore, the transmission range and link reliability are also affected by this factor, so it permits adapting the LoRa modulation to the transmission conditions of the scenario under consideration [40]. Moreover, as stated above, the SF directly impacts the transmission data-rate, hence the ToA of each transmission is affected even if the packet length remains the same. Consequently, a maximum ToA has been defined for each LoRa SF configuration. These values for the European region are shown in Table 1, which also presents the ToA values related to the three messages that comprise the EDHOC transaction (please see Figure 6) for different SF configurations. Please note that each presented ToA has been calculated by considering a fixed value for the CR (4/5) and the BW (125 kHz).

As Table 1 presents, the complete EDHOC transaction can be performed by using SF7 and SF8, which constitute the fastest LoRa data-rate configurations. In addition, by making use of SF9, the first and third messages are suitable to be transmitted, as well; hence, by using a dynamic SF-selection mechanism, this configuration may be also valid in advanced LoRaWAN systems [41]. In turn, when using the most conservative settings (SF10–SF12), the ToA of the EDHOC messages surpass the regulatory radio-band restrictions. This fact has been represented in Table 1 by marking the particular SFs that are not capable of supporting the complete EDHOC message exchange. Although higher SF values permit one to increase the robustness of the transmission link, performance results in previous works [40] have demonstrated viable scenarios for the use of these LoRa configurations (SF7–SF8). Concretely, in urban environments, the chosen value of the SF parameter does not lead to an improvement of the Packet Delivery Ratio (PDR) for transmission distances below 3 km. Therefore, the exchange of the EDHOC messages remains valid for a great number of potential scenarios especially under the umbrella of the smart cities paradigm. Moreover, the adopted IPv6 compression algorithm (SCHC) is aimed at also reducing the length of CoAP headers, as well as packet fragmentation. This improvement has not been considered in this work, but its inclusion will permit the successful performance of the EDHOC transaction at higher SF values.

## 7. Conclusions

In this work, the focus was on the security characteristics of LoRaWAN, a prominent communication technology for enabling next-generation IoT applications. Although LoRaWAN already includes a security scheme for protecting the communications between end-devices and the network server, some shortcomings have been diagnosed, mostly related to the cryptographic key-updating process. In order to provide answers to these inherent limitations of the off-the-shelf LoRaWAN security solution, different alternatives have been explored and discussed, namely IKE, DTLS and EDHOC. It was found that the most important limitation imposed by LoRaWAN is that related to the maximum length of the transmitted messages. This restriction is determined by the low data-rates provided by this communication technology and the stringent frequency-band regulations, which severely limit the time-on-air of each transmission. Therefore, considering these restrictions, the EDHOC protocol was identified as the most viable solution to enable the key-updating process due to the limited size of the messages. Thus, it was demonstrated that the EDHOC transaction, composed of three messages, can be supported by the greatest data-rate LoRa configurations, namely SF7 and SF8. Finally, a complete communication stack has been also presented for enabling the inclusion of the proposed security solution within the LoRaWAN architecture. This proposal includes an adaptation layer with the aim of enabling IPv6 communication between the LoRaWAN’s end-nodes and external networks. This adaptation layer has been implemented by means of the novel SCHC scheme, recently proposed by the IETF.

Part of our future work focuses on the experimental evaluation of the developed prototype on a real LoRaWAN deployment. In addition, several improvements to the current proposal might be considered, as well. In particular, the SCHC scheme additionally defines the compression of CoAP headers, as well as message fragmentation operations, which have not been considered in this work. By incorporating these mechanisms, the performance of the proposed solution will be improved. Moreover, among our plans, we also have the evolution of our platform to use LoRaWAN 1.1 and thus adapt our key management scheme to the new keys included in the LoRa domain to further complement the new rejoin process defined.

## Figures and Tables

**Table 1:** Time-on-Air (ToA) of EDHOC messages and maximum admissible transmission times for different LoRa SF configurations.

Spreading Factor	ToA EDHOC Frame 1 (113 B)	ToA EDHOC Frame 2 (143 B)	ToA EDHOC Frame 3 (71 B)	Maximum ToA
SF7	189.70 ms	235.78 ms	128.26 ms	399.62 ms
SF8	338.43 ms	420.35 ms	236.03 ms	707.07 ms
SF9 *	615.42 ms	738.30 ms	410.62 ms	676.83 ms
SF10 *	1107.97 ms	1353.73 ms	780.29 ms	698.37 ms
SF11 *	2461.70 ms	2953.22 ms	1642.50 ms	1560.58 ms
SF12 *	4431.87 ms	5414.91 ms	3121.15 ms	2793.47 ms

* Valid LoRaWAN configuration for supporting the EDHOC transaction.

## Abbreviations

The following abbreviations are used in this manuscript:

6LoWPAN	IPv6 over Low power Wireless Personal Area Networks
ABP	Activation By Personalization
ACE	Authorization for Constrained Environments
AppEUI	Application Identifier
AppKey	Application Key
AppSKey	Application Session Key
BW	Bandwidth
CBOR	Concise Binary Object Representation
CoAP	Constrained Application Protocol
CBOR	Concise Binary Object Representation
COSE	CBOR Object Signing and Encryption
CR	Coding Rate
CSS	Chirp Spread Spectrum
DevEUI	End-Device Identifier
DHE	Ephemeral Diffie–Hellman
DTLS	Datagram Transport Layer Security
EDHOC	Ephemeral Diffie–Hellman Over COSE
IETF	Internet Engineering Task Force
IKE	Internet Key Exchange
IKEv2	Internet Key Exchange Version 2
IPv6	Internet Protocol Version 6
IoT	Internet of Things
ISM	Industrial, Scientific and Medical radio band
IoT	Internet of Things
IPsec	Internet Protocol Security
JSON	JavaScript Object Notation
LoRa	Long Range
LoRaWAN	Long Range Wide Area Network
LP-WAN	Low Power-Wide Area Network
MAC	Medium Access layer
MQTT	Message Queuing Telemetry Transport
NwkSKey	Network Session Key
OTAA	Over-The-Air Activation
PDR	Packet Delivery Ratio
PFS	Perfect Forward Secrecy
PHY	Physical layer
PSK	Pre-Shared Key
SF	Spreading Factor
ToA	Time-on-Air
UDP	User Datagram Protocol
